# Lamisil versus clotrimazole in the treatment of vulvovaginal candidiasis

**Published:** 2013-03

**Authors:** Ali Zarei Mahmoudabadi, Mahin Najafyan, Eskandar Moghimipour, Maryam Alwanian, Zahra Seifi

**Affiliations:** 1 Department of Medical Mycology, School of Medicine, Ahvaz Jundishapur University of Medical Sciences, Ahvaz, Iran; 2 Infectious Diseases and Tropical Medicine Centre, Ahvaz Jundishapur University of Medical Sciences, Ahvaz, Iran; 3 Department of Obstetric and Genecology, School of Medicine, Ahvaz Jundishapur University of Medical Sciences, Ahvaz, Iran; 4 Department of Pharmaceutics, School of Pharmacy, Ahvaz Jundishapur University of Medical Sciences, Ahvaz, Iran

**Keywords:** Vulvovaginal candidiasis, Clotrimazole, Terbinafine, *Candida albicans*

## Abstract

**Background and Objectives:**

Vaginal candidiasis is a common disease in women during their lifetime and occurs in diabetes patients, during pregnancy and oral contraceptives users. Although several antifungals are routinely used for treatment; however, vaginal candidiasis is a challenge for patients and gynecologists. The aim of the present study was to evaluate terbinafine (Lamisil) on *Candida* vaginitis versus clotrimazole.

**Materials and Methods:**

In the present study women suspected to have vulvovaginal candidiasis were sampled and disease confirmed using direct smear and culture examination from vaginal discharge. Then, patients were randomly divided into two groups, the first group (32 cases) was treated with clotrimazole and the next (25 cases) with Lamisil. All patients were followed-up to three weeks of treatment and therapeutic effects of both antifungal were compared.

**Results:**

Our results shows that 12 (37.5%) patients were completely treated with clotrimazole during two weeks and, 6(18.8%) patients did not respond to drugs and were refereed for fluconazole therapy. Fourteen (43.8%) patients showed moderate response and clotrimazole therapy was extended for one more week. When Lamisil was administrated, 19 (76.0%) patients were completely treated with Lamisil in two weeks, and 1 (4.0%) of the patients did not respond to the drug and was refereed for fluconazole therapy. Five (20.0%) of our patients showed moderate response and Lamisil therapy was extended for one more week.

**Conclusion:**

Our results show that vaginal cream, 1% Lamisil, could be suggested as a first-line treatment in vulvovaginal candidiasis.

## INTRODUCTION

Vaginitis or vaginal candidiasis is a common fungal infection that occurs under predisposing conditions such as, diabetes, antibiotic therapy, oral contraceptive and pregnancy. Disease is a common disorder among women and nearly 75% of women suffer once in their lifetime of genital candidosis (1). In addition, about 5-10% of women suffer from recurrent vaginal candidiasis during their reproductive age (2). Vaginitis is not life threatening, however disease may have morbidities in discomfort, pain, and sexual activities. In addition, women with chronic or recurrent vaginitis represent a challenging patient population. Several factors such as stressors (3), resistant to antifungal (4) and the presence of predisposing factors (1, 5) can cause vulvovaginal relapse in women.

The majority of vaginal candidiasis cases are caused by *Candida albicans*, however non-*albicans* species were also usually associated with recurrent or chronic forms of diseases (6–8). Non-*albicans* species are usually less sensitive to azole antifungal (6, 9, 10). Several investigators believe that an increasing in vaginal infection due to non-*albicans*, (*C. glabrata*, *C. tropicalis* and *C. dubliniensis*) has been observed during recent years (9, 11). Reports show that azole resistance was detected among some *Candida* species; especially *C. glabrata* isolates (9, 12, 13). In addition long term use of antifungal drugs can cause recurrent vaginitis (2).

Terbinafine (Lamisil) is a fungicidal allylamine antifungal that is used for the treatment of several fungal infections, such as dermatophytosis (14, 15), cutaneous candidiasis (16, 17), diseases due to dimorphic (17) and saprophytic fungi (18) during last two decades. Increasing in prevalence vulvovaginal candidiasis, resistance to antifungals and new predisposing diseases are caused that new antifungals present for the treatment disease. Several antifungal drugs (clotrimazole, nystatin, miconazole, and fluconazole) are available for treatment of *Candida* vaginitis; however clotrimazole is usually prescribed for treatment. Terbinafine has wide spectrum antifungal effect against several species of yeasts especially *Candida*; however there is no more data about its effect on the vulvovaginal candidiasis. This study was carried out to compare the effect of clotrimazole (1%) and terbinafine (1%) on the treatment of vulvovaginal candidiasis.

## MATERIALS AND METHODS

### Patient's identification and clinical trial

In the present study, 110 patients suspected to have *Candida* vaginal infection were randomly examined by a gynecologist and sampled. Consent informs were also signed by all patients. Patients with pregnancy, diabetes, and immunodeficiency were excluded from this clinical trial. Samples were included two vaginal swabs, one for direct smear and another cultured on CHROMagar Candida (CHROMagar Candida^®^, France). Both samples transferred immediately to medical mycology laboratory in Ahvaz Jundishapur University of Medical Sciences. Cultured media were incubated at 37°C for 24-48h. Slides were stained by methylene blue and investigated by a light microscope (X100). Cultures were also examined for color colonies, green and pink colonies were respectively *C. albicans* and *C. glabrata*. Both *Candida* species were confirmed using germ tube, production chlamydoconidia on cornmeal agar (Difco, USA) and microscopic morphology on Sabouraud's dextrose agar, SDA, (Merck, Germany). In addition, grow at 45°C was also applied for differentiation *C. albicans* from *C. dubliniensis*. The presence of pseudohyphae and blastoconidia (yeast cells and budding cells) in direct smear and positive cultures were confirmed vaginal candidiasis in patients. Totally, based on above tests, *C. albicans* and *C. glabrata* were detected from patients samples.

Then, for the first patient group clotrimazole and Lamisil for the second patient group were prescribed by a gynecologist as double blind study. Both antifungals were prescribed as three times daily. All patients were fellow upped after one and two weeks and end point in evaluation was made by clinician view. Full response to treatment and disappear all diseases signs and symptoms were considered as complete response. Relative response to terbinafine and inefficiency to drug were considered as moderate response and no response, respectively. Clinical symptoms were considered and culture examination was also prepared when necessary.

### Drugs preparation

Vaginal cream clotrimazole (1%) was purchased from drugstore as usual prescription for the treatment of vulvovaginal candidiasis. Vaginal cream terbinafine (1%) was prepared by solubilizing the drug in propylene glycol. A second system was prepared by mixing mineral oil and trihydroxystearate at 80°C, cooling to 40°C and then adding cetyl alcohol. The oily and aqueous phases were then mixed and homogenized for 20 min. Then it was divided in prewashed and cleaned tubes consisted of 20g of the drug.

## RESULTS

In the present pilot study, 57 patients with confirmed vulvovaginal candidiasis were treated with clotrimazole and Lamisil. Mycological tests were confirmed *C. albicans* 51(89.5%) as the major causative agents of vulvovaginal candidiasis followed by *C. glabrata* 6 (10.5%). In the present study, all patients were examined and followed up by the clinician based on following criteria. When all disease signs and symptoms were disappeared (completely cured with full patients satisfaction) patients were considered as completely treated while disappearing of some signs and symptoms and relative satisfaction of patients were considered as moderate response. No clinical response to antifungal drugs and persistent of infection was considered as untreated.

In the present study 3(9.4%) of patients were completely treated by clotrimazole after one week. 23(71.9%) of patients showed moderate response that continued treatment for next week (Table 1). Only 6(18.7%) of patients remained untreated, however they continued for next week. Totally, 12(37.5%) of patients completely treated with clotrimazole during two weeks and, 6(18.8%) of patients did not response to drug and refereed for fluconazole therapy. 14(43.7%) of patients showed moderate response and one week more extended for clotrimazole therapy.

**Table 1:** The results of effect clotrimazole and fluconazole on patients during two weeks.

Antifungals	Duration	Completely treated		Moderate response		Untreated		Total	

		No	%	No	%	No	%	No	%
Clotrimazole	week 1	3	9.4	23	71.9	6	18.7	32	100
	weeks 2	9	31.0	14	48.3	6	20.7	29	100
	Total	12	37.5	14	43.7	6	18.8	32	100
Lamisil	week 1	2	8.0	22	88.0	1	4.0	25	100
	weeks 2	17	73.9	5	21.7	1	4.4	23	100
	Total	19	76.0	5	20.0	1	4.0	25	100

Our results were shown that 2(8.0%) of studied patients were completely treated by Lamisil after one week, followed by 22(88.0%) of patients showed valuable response (Table 1). Totally, 19(76.0%) of patients completely treated with Lamisil during two weeks and, 1(4.0%) of patients did not response to drug and refereed for fluconazole therapy. 5(20.0%) of our patients showed moderate response and one week more extended for Lamisil therapy.

## DISCUSSION

Several studies show that *C. albicans* is the main agent of vulvovaginal candidiasis in the world (8, 9, 11, 19–21). However this rate varies in different study, 42%(19), 53.2%(20), 83%(8), 70.8%(9), 75%(21) and 87.9% (11). In addition, few reports show that *C. glabrata* (50.4%) was the most common species among the vaginal isolates (6). In our study only six isolates of *C. glabrata* (10.5%) compared with 89.5% of *C. albicans* were recovered from vaginal samples. Our previous study showed that the prevalence of *C. albicans* in this area (Ahvaz) is 94.0% and other species of *Candida* are rare (7). Overall percentage of non-*albicans* species (10.5%) in our study was much lower than in previous reports in different cities of Iran (19, 21).

*Candida* vaginitis is one of the most frequent infections of the women during reproductive age that has high incidence. Vaginal creams or suppositories of topical azoles including butoconazole, clotrimazole, econazole lipogel, fenticonazole, ketoconazole, miconazole, omoconazole, oxiconazole, and terconazole are commonly used for the treatment of vulvovaginal candidiasis. In addition a single dose of fluconazole (150 to 300 mg) can also be used (22). Several reports show that resistant to fluconazole is different in literatures. Mohanty *et al*. (6) believe that there is a low prevalence of fluconazole resistance in vaginal *Candida* in India. Whereas the recurrence rate of symptomatic vaginitis by non-*albicans* species after fluconazole therapy was 50.4% and 54.2% at 6 months at 12 months in the USA (10).

Probiotics therapy (lactobacilli therapy) is recently used for the treatment of vulvovaginal candidiasis (2). Although several anti-*Candida* drugs are available for the treatment of vulvovaginal candidiasis, there are reports show that recurrent and resistance to antifungals are more prevalent among patients (2, 23). In addition, in the study of Richter *et al*. (9), 3.6% and 16.2% vaginal isolates of *C. albicans* were resistant to fluconazole and itraconazole, respectively. They were also reported that econazole, clotrimazole, miconazole, and ketoconazole were active against 94.3% to 98.5% of isolates at <1µg/ml. Terbinafine is a broad spectrum and effective antifungal drug of allylamine class. It has fungicidal activity against several fungi, dermatophytes, moulds and certain dimorphic fungi and fungistatic activity against yeasts. Oral administration of terbinafine is absorbed rapidly and distributed in the stratum corneum, sebum, nails and hair (17).

There are a few reports that show effect Lamisil on vulvovaginal candidiasis or vaginitis agent (24). Ferahbas *et al*. (24) in a clinical trial were compared the effect of three orally antifungals, terbinafine, itraconazole, and fluconazole in patients with vulvovaginal candidiasis. They revealed an overall cure rate (clinical and mycologic cure rates) 33.3% for terbinafine, 10% for itraconazole, and 53.3% for fluconazole. As results they were believed that terbinafine could be an alternative for the treatment of vulvovaginal candidiasis. In contrast, our study shows that clinical and mycologic cure rates after two weeks were 96% and 81.2% for Lamisil and clotrimazole, respectively.

## CONCLUSION

Our results show that Lamisil vaginal cream (1%) could be suggested as a first-line treatment in vulvovaginal candidiasis. However, use of drug in larger numbers of cases with different *Candida* species and several types of vulvovaginal candidiasis (chronic, recurrent and acute) may give more data about the suitable effectiveness of Lamisil.
